# The regulatory landscape of RIF-mediated ripening control in strawberry

**DOI:** 10.1093/plcell/koad225

**Published:** 2023-08-22

**Authors:** Humberto Herrera-Ubaldo

**Affiliations:** Assistant Features Editor, The Plant Cell, American Society of Plant Biologists; Department of Plant Sciences, University of Cambridge, Cambridge CB2 3EA, UK

The final stage of fruit development, ripening, involves major changes in size, coloration, and textures. The popular fruit crop strawberry turns red and develops a characteristic aroma during ripening. In addition, enzymes that modify matrix glycans and pectins induce cell wall disassembly, giving the strawberry a favorable texture (Chen et al. 2020). The molecular regulation of strawberry fruit ripening is a complex process that is tightly controlled at the transcript level and involves the action of numerous transcription factors (TFs), including MYB10, MYB1, FaEOBII, and FaDOF2 (Sánchez-Gómez et al. 2022). However, our knowledge of all the molecular players and their hierarchy in this regulatory network is incomplete.

Recent work identified *RIPENING INDUCING FACTOR* (*FaRIF*), a NAC transcription factor that plays a role in controlling ripening-related processes in the octoploid strawberry *Fragaria × ananassa*, such as color, the accumulation of sugars and organic acids, and fruit softening (Martin-Pizarro et al. 2021). In this issue, **Xiaojing Li and colleagues** (Li et al. 2023) expand the regulatory landscape of RIF-mediated ripening control by identifying regulatory elements acting down- and upstream of RIF, highlighting its role as a key regulator of fruit ripening. Using genome editing, the authors generated *rif* knockout lines in the diploid strawberry *Fragaria vesca*. The CRISPR-Cas9–generated *Fvrif-6* and *Fvrif-13* lines displayed a failure in the ripening process (see Figure), as demonstrated by reduced anthocyanin levels, sugar content, and altered softening. On the contrary, the overexpression of *FvRIF* (*FvRIF*-OE lines) accelerated the ripening process.

**Figure.**
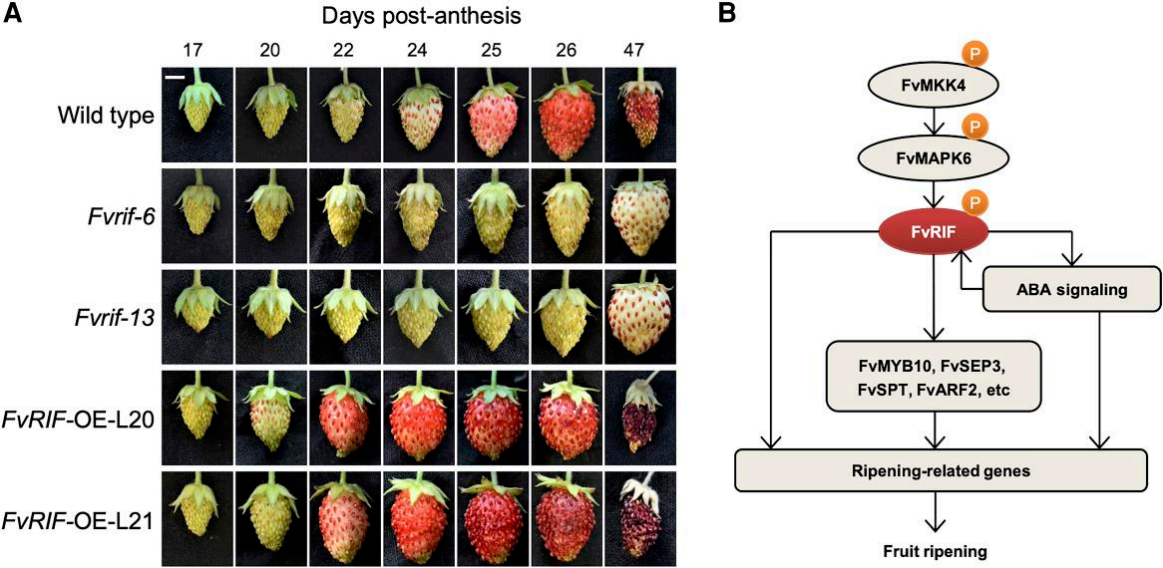
Regulation of strawberry ripening by FvRIF. **A)** Strawberry ripening process in wild type, *RIPENING INDUCING FACTOR* knockout (*Rvrif*), and overexpression (*RvRIF*-OE) lines in *Fragaria vesca*. **B)** A proposed model for regulation of fruit ripening by FvRIF. Bar represents 0.5 cm. Adapted from Li et al. (2023), Figures 1 and 10.

To identify genes acting downstream of FvRIF, the authors conducted DNA affinity purification and sequencing and transcriptomic analyses of the knockout lines. Around 8,000 FvRIF-bound sequences were identified using DNA affinity purification and sequencing. Among the 6,000 differentially expressed genes of the transcriptomic analyses, more than 2,000 differentially expressed genes harbor FvRIF binding sites, suggesting direct regulation by FvRIF. These direct targets are associated with major biochemical pathways of the strawberry ripening process, such as anthocyanin biosynthesis that determines color changes (*CHALCONE SYNTHASE*, *DIHYDROFLAVONOL-4-REDUCTASE*, *ANTHOCYANIDIN SYNTHASE*, and *UDP-GLUCOSE FLAVONOID GLUCOSYL-TRANSFERASE*) and fruit softening regulated by enzymes acting on the cell wall (*PECTATE LYASE2*, *POLYGALACTURONASE2*, *EXPANSIN3*, and *XYLOGLUCAN ENDOTRANSGLUCOSYLASE/HYDROLASE*). Additionally, FvRIF regulates the expression of other TFs, including *MYB10*, *SEPALLATA3*, *SPATULA*, and the *AUXIN RESPONSE FACTOR2*, previously identified as fruit ripening regulators (Sánchez-Gómez et al. 2022).

The authors also explored the upstream regulation of FvRIF activity. Some features and functions of TFs, such as subcellular localization, transcriptional activity, and protein-protein interactions, are modulated by posttranslational modifications. Li and colleagues focused on protein phosphorylation because the FvRIF protein sequence has 5 putative phosphorylation sites. They tested the physical interaction between FvRIF and the 12 mitogen-activated protein kinases (MAPKs) in the *F. vesca* genome. They found a physical interaction with MAPK6, and the colocalization of the proteins suggested it is a functionally relevant interaction. Protein phosphorylation assays confirmed FvRIF as a target of MAPK6. The authors used FvMKK4 as an activator of MAPK6 since a recent study identified it as the activator of FvMAPK3 (Mao et al. 2022). Site-directed mutagenesis revealed that the threonine at position 310 (T310) is critical for FvRIF function. The T310 to alanine (T310A) substitution altered the expression patterns of selected target genes, and complementation of the *Fvrif-13* knockout line by FvRIF^T310A^ failed to recover the wild-type phenotype, indicating that the specific phosphorylation of FvRIF at T310 by MAPK6 is critical for strawberry ripening.

This work expands our understanding of the regulatory landscape of RIF-mediated ripening control and is a step forward toward engineering improvements in fruit quality.

## Dive Curated Terms

The following phenotypic, genotypic, and functional terms are of significance to the work described in this paper:

MAPK6 Gramene: AT2G43790
MAPK6 Araport: AT2G43790

ANTHOCYANIDIN CHEBI: CHEBI:38695

CHALCONE CHEBI: CHEBI:27618

NAC CHEBI: CHEBI:7421

PECTATE CHEBI: CHEBI:68837

XYLOGLUCAN CHEBI: CHEBI:18233

protein CHEBI: CHEBI:36080

MYB10 Gramene: FvH4_1g22020
MYB10 Araport: FvH4_1g22020
